# Supplementary material for: Exposure of gut bacterial isolates to the anthelminthic drugs, ivermectin and moxidectin, leads to antibiotic-like phenotypes of growth inhibition and adaptation
Source: Commun Biol. 2024 Nov 25;7:1566. doi: 10.1038/s42003-024-07135-z (PMC11603213; doi:10.1038/s42003-024-07135-z)
Supplement: Supplementary file 2 — Supplementary Information [file 42003_2024_7135_MOESM2_ESM.pdf]

Supplementary Figures and Tables concerning the manuscript:

Exposure of gut bacterial isolates to two widely used anthelminthic drugs, ivermectin and moxidectin, leads to antibiotic-like phenotypes of growth inhibition and adaptation.

Julian Dommann[a,b], Jennifer Keiser[a,b], Julian Garneau[c], Alison Gandelin[c], Carlo Casanova[d], Peter M. Keller[e], Somphou Sayasone[a,b,f], Pascale Vonaesch[c], Pierre H. H. Schneeberger[a,b]*

[a]Swiss Tropical and Public Health Institute, Department of Medical Parasitology and Infection Biology, Allschwil, Switzerland.

[b]University of Basel, Basel, Switzerland.

[c]Department of Fundamental Microbiology, University of Lausanne, Lausanne, Switzerland.

[d]Institute for Infectious Diseases, University of Bern, Bern, Switzerland

[e]Clinical Bacteriology and Mycology, University Hospital Basel, Basel, Switzerland

[f]Lao Tropical and Public Health Institute, Ministry of Health, Vientiane, Lao People's Democratic Republic

Supplementary Table 1: Overview of the 20 macrolide (erythromycin) or lincosamide (clindamycin) resistant bacterial isolates used in the study.

isolate	sample type	resistance (MIC[mg/l])
Actinomyces naeslundii (01)	blood cluture	clindamycin (>256)
Actinomyces odontolyticus (01)	wound swab (cervical)	clindamycin (>256)
Actinomyces odontolyticus (02)	peritonsillar abscess swab	clindamycin (>256)
Streptococcus dysgalactiae (01)	synovial fluid knee	clindamycin (>256)
Streptococcus equi (01)	periprosthetic tissue biopsy	clindamycin (1.5)
Streptococcus mitis (01)	blood culture	clindamycin (0.19)
Streptococcus oralis (01)	blood culture	clindamycin (>256)
Streptococcus oralis (02)	blood culture	clindamycin (>256)
Streptococcus pneumoniae (01)	blood culture	erythromycin
Streptococcus pneumoniae (02)	blood culture	erythromycin
Streptococcus pneumoniae (03)	blood culture	erythromycin
Streptococcus pneumoniae (04)	blood culture	erythromycin
Streptococcus pneumoniae (05)	blood culture	erythromycin
Streptococcus pneumoniae (06)	blood culture	erythromycin
Streptococcus pneumoniae (07)	blood culture	erythromycin
Streptococcus pneumoniae (08)	blood culture	erythromycin
Streptococcus pneumoniae (09)	blood culture	erythromycin
Streptococcus pneumoniae (10)	blood culture	erythromycin
Streptococcus pneumoniae (11)	blood culture	erythromycin
Streptococcus pneumoniae (12)	blood culture	erythromycin

Supplementary Table 2: Overview of the 27 clinical bacterial isolates used in the study (UNIL = University of Lausanne, Lausanne Switzerland; SwissTPH = Swiss Tropical and Public Health Institute, Allschwil, Switzerland).

isolate	enrichment site	country of origin	sample type	enrichment media
Blautia luti (01)	UNIL	Lao PDR	stool	mGAM
Clostridium baratii (01)	UNIL	Lao PDR	stool	Schaedler
Clostridium baratii (02)	UNIL	Lao PDR	stool	mGAM
Clostridium perfringens (01)	UNIL	Lao PDR	stool	BHI + inulin
Clostridium perfringens (02)	UNIL	Lao PDR	stool	BHI + inulin
Enterococcus faecalis (01)	SwissTPH	Lao PDR	stool	TH
Enterococcus faecalis (02)	SwissTPH	Lao PDR	stool	LB
Enterococcus faecalis (03)	SwissTPH	Lao PDR	stool	TH
Enterococcus faecium (01)	SwissTPH	Lao PDR	stool	BHI + 5% yeast
Enterococcus faecium (02)	SwissTPH	Lao PDR	stool	LB
Enterococcus faecium (03)	SwissTPH	Lao PDR	stool	mGAM
Enterococcus hirae (01)	SwissTPH	Lao PDR	stool	BHI + 5% yeast
Enterococcus hirae (02)	SwissTPH	Lao PDR	stool	BHI + 5% yeast
Enterococcus hirae (03)	SwissTPH	Lao PDR	stool	BHI + 5% yeast
Enterococcus hirae (04)	SwissTPH	Lao PDR	stool	BHI + 5% yeast
Enterococcus hirae (05)	SwissTPH	Lao PDR	stool	BHI + 5% yeast
Enterococcus hirae (06)	SwissTPH	Lao PDR	stool	TH
Escherichia coli (01)	SwissTPH	Lao PDR	stool	LB
Escherichia coli (02)	SwissTPH	Lao PDR	stool	TH

Escherichia coli (03)	UNIL	Lao PDR	stool	mGAM
Escherichia coli (04)	UNIL	Lao PDR	stool	BHI + inulin
Escherichia coli (05)	UNIL	Lao PDR	stool	Schaedler
Streptococcus anginosus (01)	SwissTPH	Lao PDR	stool	TH
Streptococcus equinus (01)	SwissTPH	Lao PDR	stool	BHI + 5% yeast
Streptococcus equinus (02)	SwissTPH	Lao PDR	stool	mGAM
Streptococcus lutetiensis (01)	SwissTPH	Lao PDR	stool	BHI + 5% yeast
Streptococcus sanguinis(01)	UNIL	Lao PDR	stool	mGAM

Supplementary Table 3: Overview of the 12 commercial bacterial isolates used in the study (UNIL = University of Lausanne, Lausanne Switzerland).

**isolate**	**strain designation**	**source**
Bacteroides stercoris (01)	DSM 19555	Ordered
Bacteroides uniformis (01)	DSM 6597	Ordered
Blautia obeum (01)	DSM 25238	Provided by UNIL
Blautia luti (02)	DSM 19850	Provided by UNIL
Blautia luti (03)	DSM 25403	Provided by UNIL
Dorea formicigenerans (01)	JCM 31256	Provided by UNIL
Dorea formicigenerans (02)	DSM 3992	Ordered
Dorea longicatena (01)	DSM 13814	Ordered
Lactobacillus salivarius (01)	DSM 20555	Ordered
Staphylococcus aureus (01)	DSM 20231	Ordered
Streptococcus parasanguinis (01)	DSM 6778	Ordered
Streptococcus salivarius (01)	DSM 20067	Ordered

Supplementary Table 4: Overview of the 10 challenged isolates used in the study (IV = ivermectin, MX = moxidectin, P = "number of passages", i.e. the number of times the bacterial culture was cultivated under the specified growth conditions for a duration of G. G = "generation time", i.e. the duration for which the bacterial culture was left to grow under the specified growth conditions. B = "bottleneck", i.e. the volume of starting culture that was used to initiate a new bacterial culture in higher concentration of either IV or MX). For S. pneumoniae (02-MXc) and S. mitis (01-MXc), the highest moxidectin concentration that still yielded growth was 10µM. All other isolates grew in presence of 20µM IV or MX.

**original isolate**	**5µM**				**10µM**				**20µM**				**challenged isolate**
	**drug**	**P1**	**G1**	**B1**	**drug**	**P2**	**G2**	**B2**	**drug**	**P3**	**G3**	**B3**	
S. salivarius (01)	IV	1	24h	10ul	IV	1	24h	10ul	IV	1	24h	10ul	*S. salivarius (01-IVc)*
S. salivarius (01)	MX	1	24h	10ul	MX	1	24h	10ul	MX	1	24h	10ul	*S. salivarius (01-MXc)*
S. parasanguinis (01)	IV	1	24h	10ul	IV	1	24h	10ul	IV	1	24h	10ul	*S. parasanguinis (01-IVc)*
S. parasanguinis (01)	MX	1	24h	10ul	MX	1	24h	10ul	MX	1	24h	10ul	*S. parasanguinis (01-MXc)*
S. pneumoniae (02)	IV	1	24h	10ul	IV	2	48h	10ul	IV	2	24h	10ul	*S. pneumoniae (02-IVc)*
S. pneumoniae (02)	MX	1	24h	10ul	MX	2	48h	10ul	-	-	-	-	*S. pneumoniae (02-MXc)*
S. mitis (01)	IV	1	24h	10ul	IV	1	24h	10ul	IV	1	24h	10ul	*S. mitis (01-IVc)*
S. mitis (01)	MX	1	24h	10ul	MX	2	48h	10ul	-	-	-	-	*S. mitis (01-MXc)*
S. dysgalactiae (01)	IV	1	24h	10ul	IV	1	24h	10ul	IV	1	24h	10ul	*S. dysgalactiae (01-IVc)*
S. dysgalactiae (01)	MX	1	24h	10ul	MX	1	24h	10ul	MX	1	48h	10ul	*S. dysgalactiae (01-MXc)*

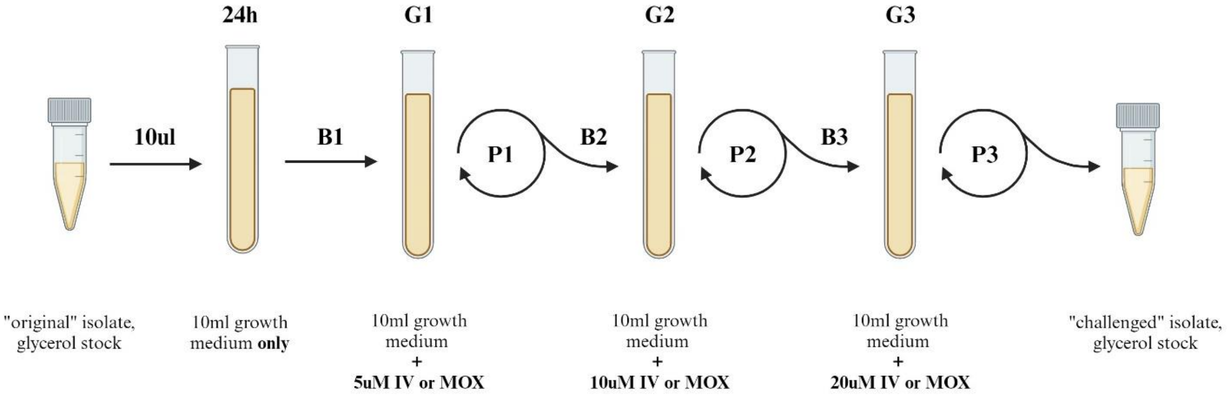

Supplementary Figure 1: Overview of the anthelminthic challenging experiment. (IV = ivermectin, MX = moxidectin, P = "number of passages", i.e. the number of times the bacterial culture was cultivated under the specified growth conditions for a duration of G. G = "generation time", i.e. the duration for which the bacterial culture was left to grow under the specified growth conditions. B = "bottleneck", i.e. the volume of starting culture that was used to initiate a new bacterial culture in higher concentration of either IV or MX). Created with BioRender.com.

Supplementary Table 5: Pairwise Wilcoxon rank sum test of AUC ratios for 11 co-incubations in presence of anthelminthics (IV = ivermectin, MX = moxidectin) and antibiotics (EM = erythromycin, CH = clarithromycin, AZ = azithromycin, CL = clindamycin, CX = ciprofloxacin, TC = tetracycline, MP = meropenem, IP = imipenem) at 1µM.

Pairwise Wilcoxon rank sum test (1µM)

	AZ	CH	CL	CX	EM	IP	IV	MP	MX
**CH**	0.086	-							
**CL**	0.147	0.918	-						
**CX**	0.000	0.000	0.000	-					
**EM**	0.329	0.248	0.393	0.000	-				
**IP**	0.031	0.076	0.159	0.000	0.035	-			
**IV**	0.000	0.000	0.000	0.749	0.000	0.001	-		
**MP**	0.035	0.114	0.303	0.001	0.067	0.886	0.001	-	
**MX**	0.000	0.000	0.000	0.632	0.000	0.000	0.665	0.001	-
**TC**	0.949	0.248	0.159	0.006	0.463	0.031	0.006	0.049	0.011

Supplementary Table 6: Pairwise Wilcoxon rank sum test of AUC ratios for 11 co-incubations in presence of anthelminthics (IV = ivermectin, MX = moxidectin) and antibiotics (EM = erythromycin, CH = clarithromycin, AZ = azithromycin, CL = clindamycin, CX = ciprofloxacin, TC = tetracycline, MP = meropenem, IP = imipenem) at 5µM.

Pairwise Wilcoxon rank sum test (5µM)

	AZ	CH	CL	CX	EM	IP	IV	MP	MX
**CH**	0.025	-							
**CL**	0.531	0.409	-						
**CX**	0.030	0.005	0.014	-					
**EM**	0.249	0.290	0.787	0.005	-				
**IP**	0.005	0.187	0.086	0.004	0.025	-			
**IV**	0.753	0.066	0.235	0.187	0.183	0.014	-		
**MP**	0.011	0.380	0.149	0.004	0.086	0.970	0.025	-	
**MX**	0.939	0.086	0.667	0.086	0.165	0.025	0.875	0.029	-
**TC**	0.908	0.352	0.718	0.187	0.702	0.066	0.718	0.086	1.000

Supplementary Table 7: Pairwise Wilcoxon rank sum test of AUC ratios for 11 co-incubations in presence of anthelminthics (IV = ivermectin, MX = moxidectin) and antibiotics (EM = erythromycin, CH = clarithromycin, AZ = azithromycin, CL = clindamycin, CX = ciprofloxacin, TC = tetracycline, MP = meropenem, IP = imipenem) at 10µM.

Pairwise Wilcoxon rank sum test (10µM)									
	**AZ**	**CH**	**CL**	**CX**	**EM**	**IP**	**IV**	**MP**	**MX**
**CH**	0.161	**-**							
**CL**	0.870	0.266	**-**						
**CX**	0.632	0.113	0.477	**-**					
**EM**	0.287	0.584	0.619	0.220	**-**				
**IP**	0.035	0.657	0.220	0.035	0.266	**-**			
**IV**	0.657	0.550	0.904	0.517	0.657	0.310	**-**		
**MP**	0.035	0.657	0.220	0.035	0.266	0.949	0.266	**-**	
**MX**	0.266	0.949	0.517	0.220	0.657	0.657	0.657	0.657	**-**
**TC**	0.657	0.508	0.745	0.632	0.870	0.266	0.937	0.266	0.657

Supplementary Table 8: Pearson correlation matrix of AUC ratios for 11 co-incubations in presence of anthelminthics (IV = ivermectin, MX = moxidectin) and macrolide/lincosamide antibiotics (EM = erythromycin, CH = clarithromycin, AZ = azithromycin, CL = clindamycin) at 1µM. Values in bold are different from 0 with a significance level alpha=0.05.

Variables	IV	MX	EM	CH	AZ	CL
IV	**1**	**0.747**	0.349	0.493	0.410	0.096
MX	**0.747**	**1**	0.518	**0.756**	0.575	0.088
EM	0.349	0.518	**1**	**0.920**	**0.954**	0.359
CH	0.493	**0.756**	**0.920**	**1**	**0.926**	0.203
AZ	0.410	0.575	**0.954**	**0.926**	**1**	0.207
CL	0.096	0.088	0.359	0.203	0.207	**1**

Supplementary Table 9: Pearson correlation p-values of AUC ratios for 11 co-incubations in presence of anthelminthics (IV = ivermectin, MX = moxidectin) and macrolide/lincosamide antibiotics (EM = erythromycin, CH = clarithromycin, AZ = azithromycin, CL = clindamycin) at 1µM.

Variables	IV	MX	EM	CH	AZ	CL
IV	**0**	**0.008**	0.293	0.124	0.210	0.779
MX	**0.008**	**0**	0.103	**0.007**	0.064	0.798
EM	0.293	0.103	**0**	**<0.0001**	**<0.0001**	0.278
CH	0.124	**0.007**	**<0.0001**	**0**	**<0.0001**	0.549
AZ	0.210	0.064	**<0.0001**	**<0.0001**	**0**	0.541
CL	0.779	0.798	0.278	0.549	0.541	**0**

Supplementary Table 10: Pearson correlation matrix of AUC ratios for 11 co-incubations in presence of anthelminthics (IV = ivermectin, MX = moxidectin) and macrolide/lincosamide antibiotics (EM = erythromycin, CH = clarithromycin, AZ = azithromycin, CL = clindamycin) at 5µM. Values in bold are different from 0 with a significance level alpha=0.05.

Variables	IV	MX	EM	CH	AZ	CL
IV	**1**	0.322	**0.647**	0.590	**0.826**	0.150
MX	0.322	**1**	**0.608**	0.595	0.285	0.337
EM	**0.647**	**0.608**	**1**	**0.957**	**0.792**	0.426
CH	0.590	0.595	**0.957**	**1**	**0.639**	0.381
AZ	**0.826**	0.285	**0.792**	**0.639**	**1**	0.163
CL	0.150	0.337	0.426	0.381	0.163	**1**

Supplementary Table 11: Pearson correlation p-values of AUC ratios for 11 co-incubations in presence of anthelminthics (IV = ivermectin, MX = moxidectin) and macrolide/lincosamide antibiotics (EM = erythromycin, CH = clarithromycin, AZ = azithromycin, CL = clindamycin) at 5µM.

Variables	IV	MX	EM	CH	AZ	CL
IV	**0**	0.334	**0.032**	0.056	**0.002**	0.660
MX	0.334	**0**	**0.047**	0.053	0.396	0.310
EM	**0.032**	**0.047**	**0**	**<0.0001**	**0.004**	0.192
CH	0.056	0.053	**<0.0001**	**0**	**0.034**	0.247
AZ	**0.002**	0.396	**0.004**	**0.034**	**0**	0.632
CL	0.660	0.310	0.192	0.247	0.632	**0**

Supplementary Table 12: Pearson correlation matrix of AUC ratios for 11 co-incubations in presence of anthelminthics (IV = ivermectin, MX = moxidectin) and macrolide/lincosamide antibiotics (EM = erythromycin, CH = clarithromycin, AZ = azithromycin, CL = clindamycin) at 10µM. Values in bold are different from 0 with a significance level alpha=0.05.

Variables	IV	MX	EM	CH	AZ	CL
IV	**1**	0.170	**0.651**	**0.731**	**0.706**	0.155
MX	0.170	**1**	0.475	0.529	0.231	0.253
EM	**0.651**	0.475	**1**	**0.923**	**0.913**	0.449
CH	**0.731**	0.529	**0.923**	**1**	**0.800**	0.363
AZ	**0.706**	0.231	**0.913**	**0.800**	**1**	0.426
CL	0.155	0.253	0.449	0.363	0.426	**1**

Supplementary Table 13: Pearson correlation p-values of AUC ratios for 11 co-incubations in presence of anthelminthics (IV = ivermectin, MX = moxidectin) and macrolide/lincosamide antibiotics (EM = erythromycin, CH = clarithromycin, AZ = azithromycin, CL = clindamycin) at 10µM.

Variables	IV	MX	EM	CH	AZ	CL
IV	**0**	0.618	**0.030**	**0.011**	**0.015**	0.650
MX	0.618	**0**	0.140	0.094	0.494	0.452
EM	**0.030**	0.140	**0**	**<0.0001**	**<0.0001**	0.166
CH	**0.011**	0.094	**<0.0001**	**0**	**0.003**	0.272
AZ	**0.015**	0.494	**<0.0001**	**0.003**	**0**	0.191
CL	0.650	0.452	0.166	0.272	0.191	**0**

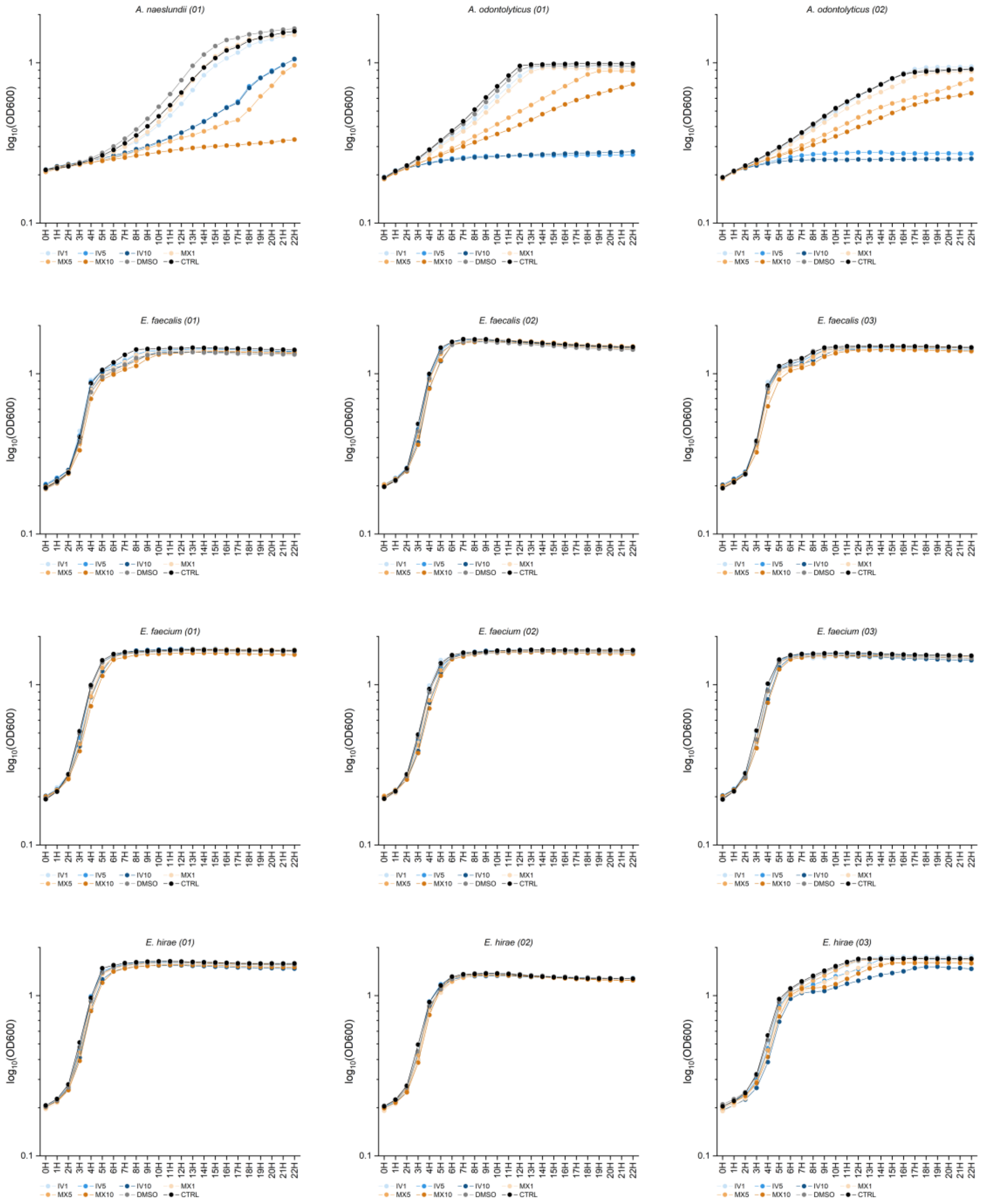

Supplementary Figure 2: Aerobic growth curves of 12 bacterial isolates co-incubated with IV (blue) or MX (orange) at concentrations of either 1µM, 5µM or 10µM. Each datapoint represents an average of two independent experiments. The grey curves represent growth in presence of 0.2% DMSO, the black curve represents a positive growth control in presence of BHI + 5% yeast only. Left to right and top to bottom: A. naeslundii (01), A. odontolyticus (01), A. odontolyticus (02), E. faecalis (01), E. faecalis (02), E. faecalis (03), E. faecium (01), E. faecium (02), E. faecium (03), E. hirae (01), E. hirae (02) and E. hirae (03).

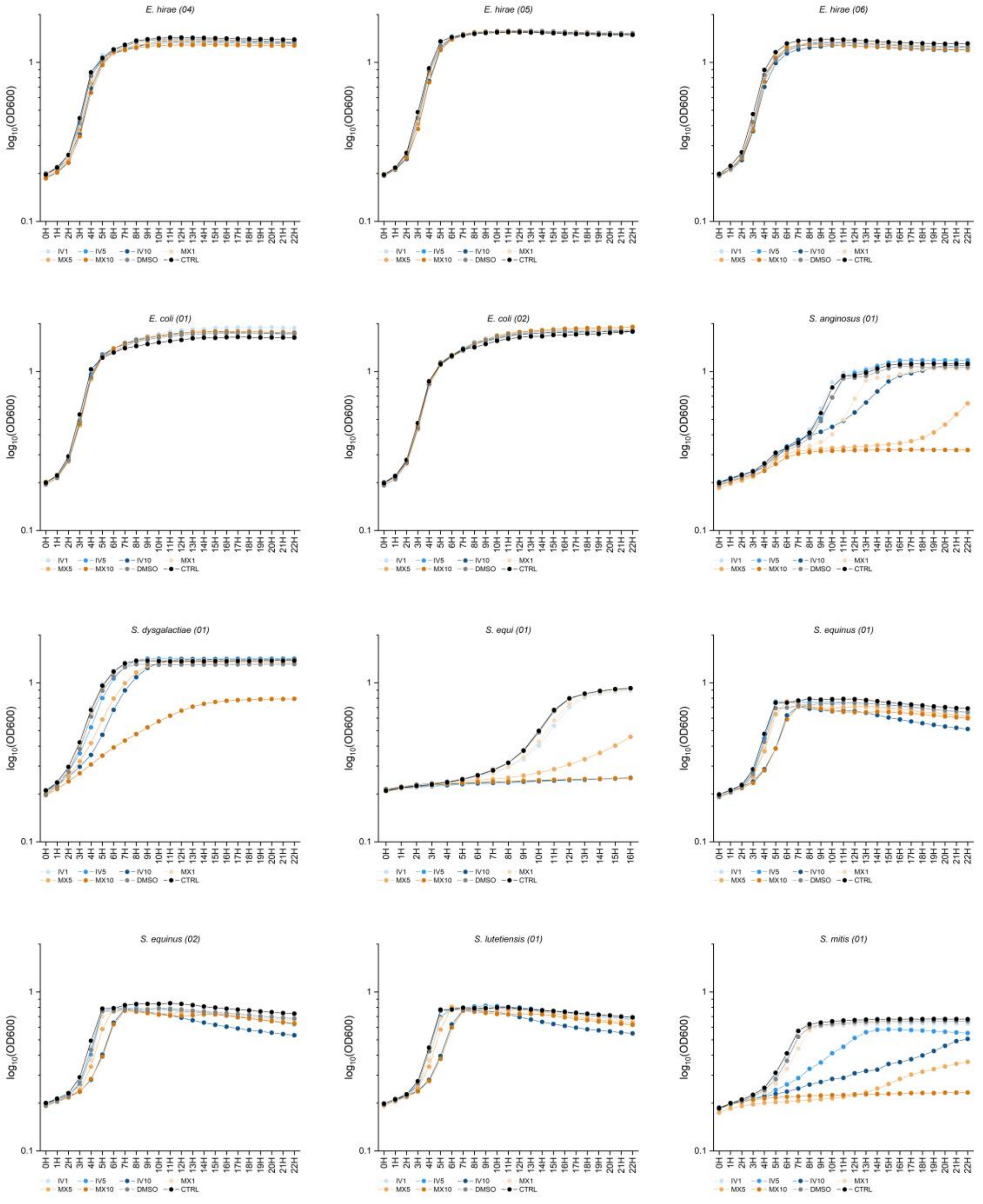

Supplementary Figure 3: Aerobic growth curves of 12 bacterial isolates co-incubated with IV (blue) or MX (orange) at concentrations of either 1µM, 5µM or 10µM. Each datapoint represents an average of two independent experiments. The grey curves represent growth in presence of 0.2% DMSO, the black curve represents a positive growth control in presence of BHI + 5% yeast only. Left to right and top to bottom: E. hirae (04), E. hirae (05), E. hirae (06), E. coli (01), E. coli (02), S. anginosus (01), S. dysgalactiae (01), S. equi (01), S. equinus (01), S. equinus (02), S. lutetiensis (01) and S. mitis (01).

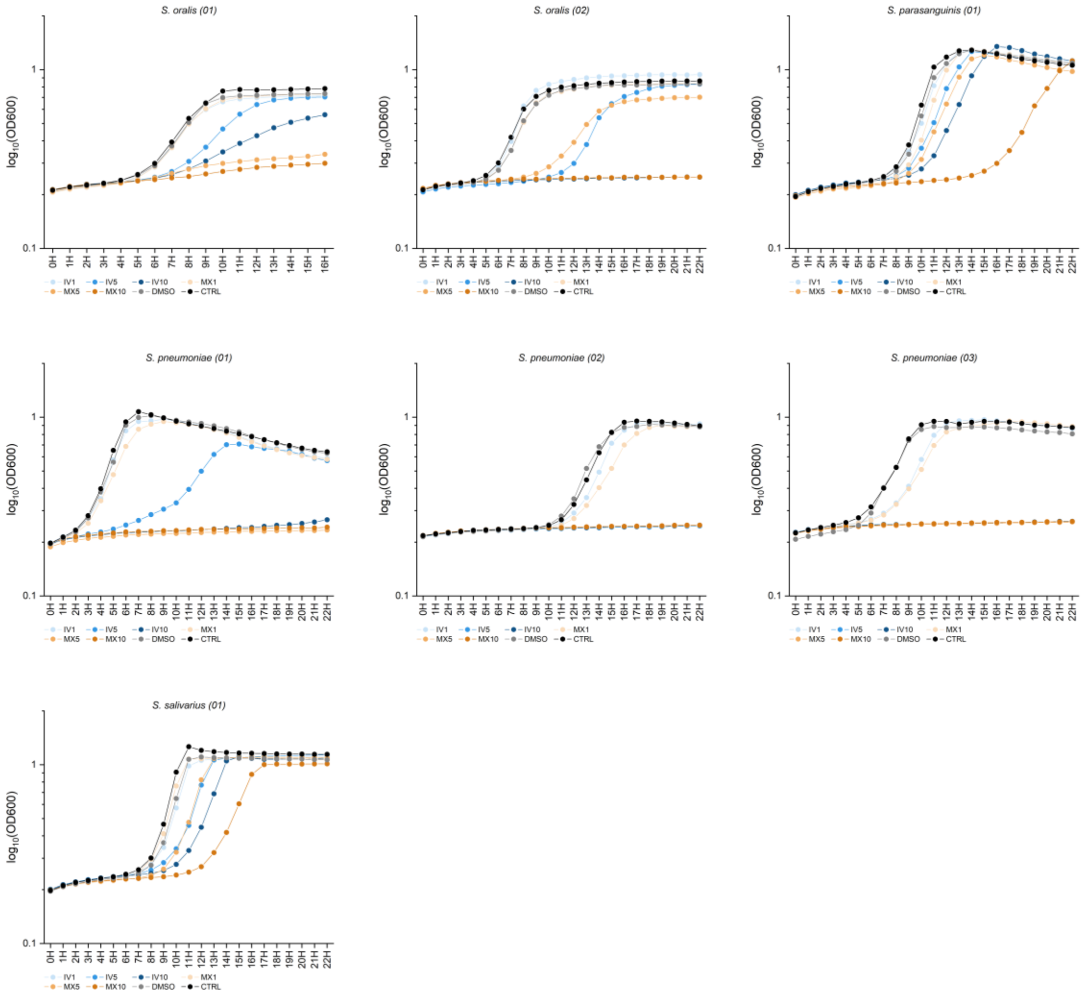

Supplementary Figure 4: Aerobic growth curves of 7 bacterial isolates co-incubated with IV (blue) or MX (orange) at concentrations of either 1µM, 5µM or 10µM. Each datapoint represents an average of two independent experiments. The grey curves represent growth in presence of 0.2% DMSO, the black curve represents a positive growth control in presence of BHI + 5% yeast only. Left to right and top to bottom: S. oralis (01), S. oralis (02), S. parasanguinis (01), S. pneumoniae (01), S. pneumoniae (02), S. pneumoniae (03) and S. salivarius (01).

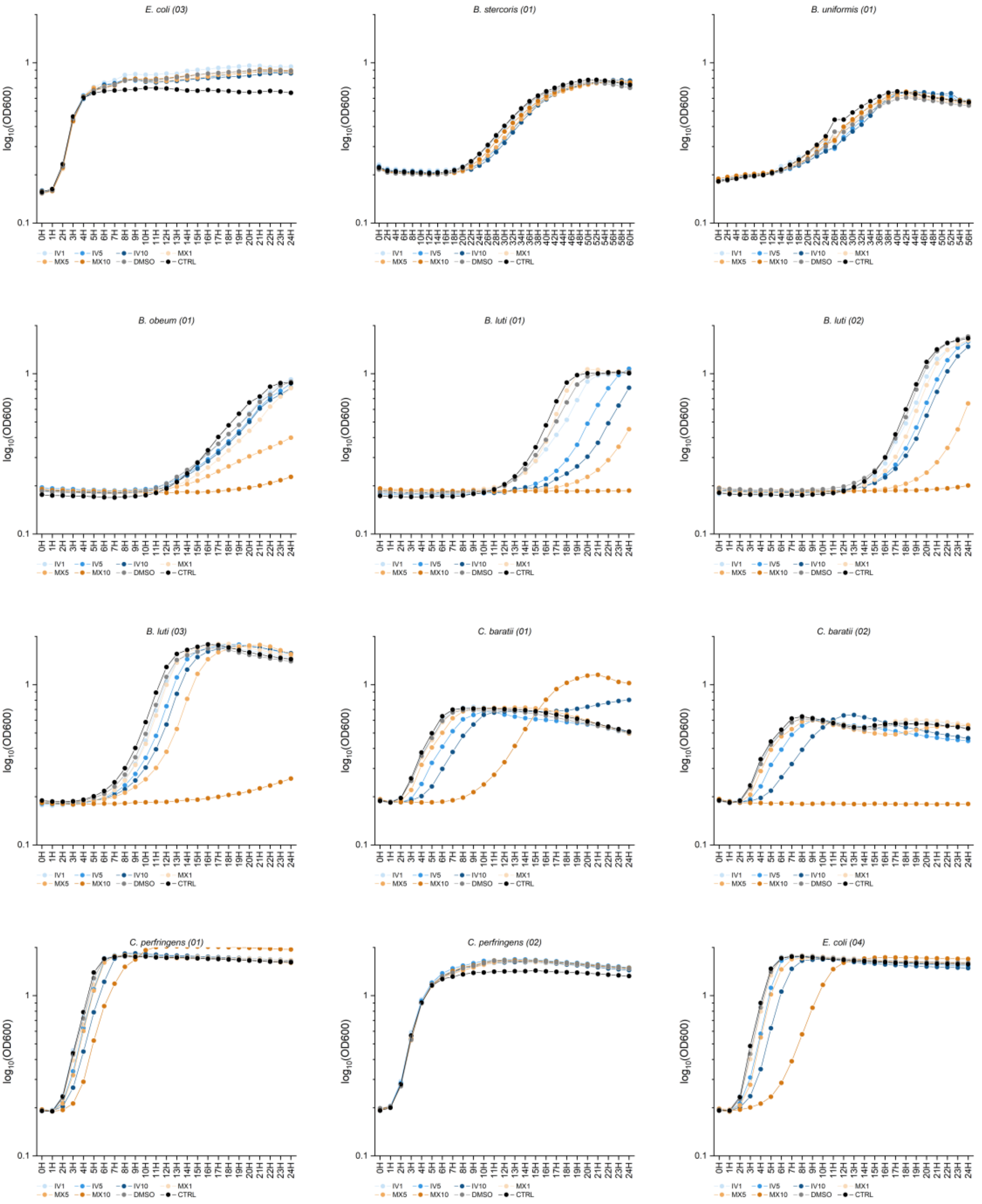

Supplementary Figure 5: Anaerobic growth curves of 12 bacterial isolates co-incubated with IV (blue) or MX (orange) at concentrations of either 1µM, 5µM or 10µM. Each datapoint represents an average of two independent experiments. The grey curves represent growth in presence of 0.2% DMSO, the black curve represents a positive growth control in presence of BHI + 5% yeast only. Left to right and top to bottom: E. coli (03), B. stercoris (01), B. uniformis (01), B. obeum (01), B. luti (01), B. luti (02), B. luti (03), C. baratii (01), C. baratii (02), C. perfringens (01), C. perfringens (02) and E. coli (04).

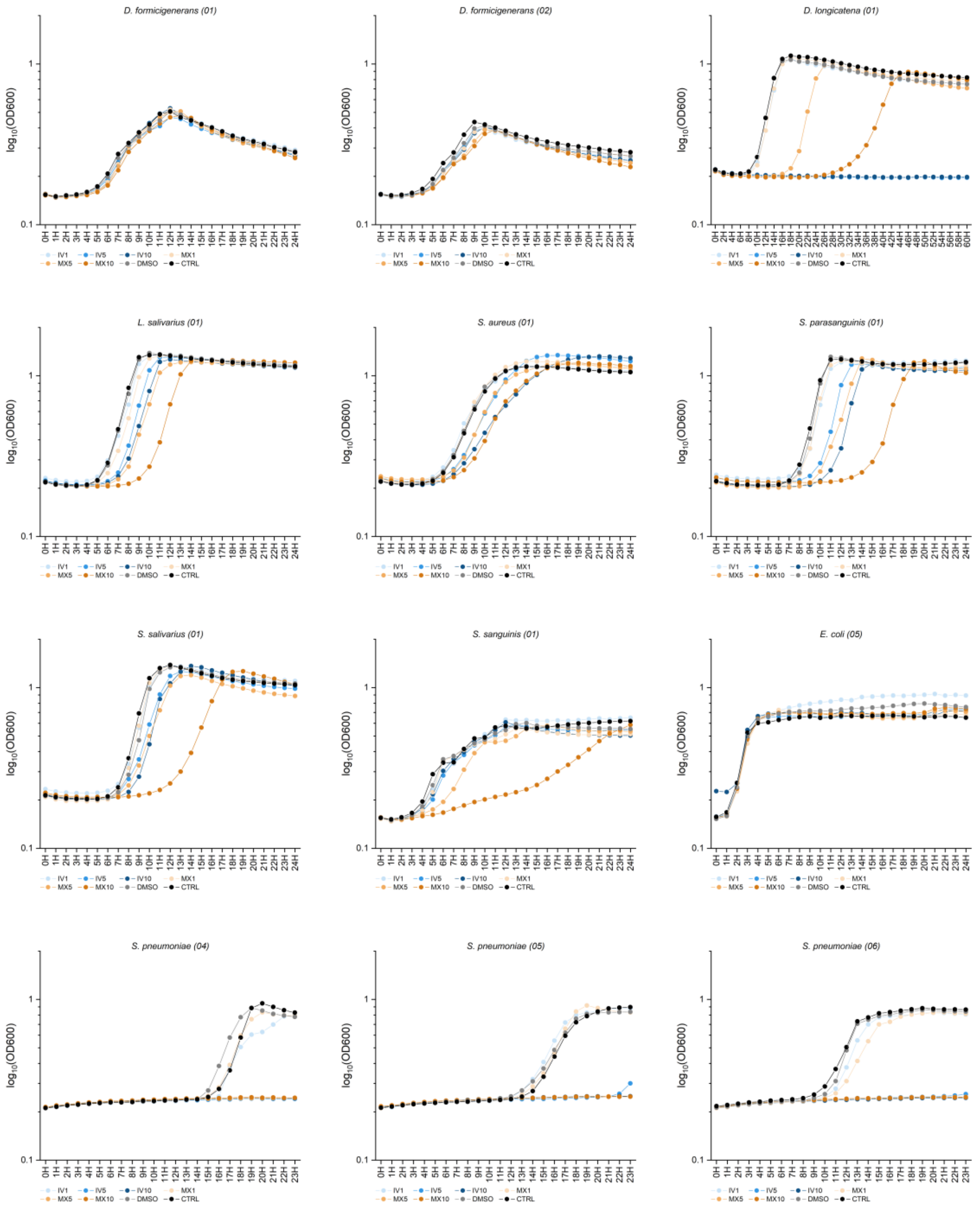

Supplementary Figure 6: Anaerobic growth curves of 12 bacterial isolates co-incubated with IV (blue) or MX (orange) at concentrations of either 1µM, 5µM or 10µM. Each datapoint represents an average of two independent experiments. The grey curves represent growth in presence of 0.2% DMSO, the black curve represents a positive growth control in presence of BHI + 5% yeast only. Left to right and top to bottom: D. formicigenerans (01), D. formicigenerans (02), D. longicatena (01), L. salivarius (01), S. aureus (01), S. parasanguinis (01), S. salivarius (01), S. sanguinis (01), E. coli (05), S. pneumoniae (04), S. pneumoniae (05) and S. pneumoniae (06).

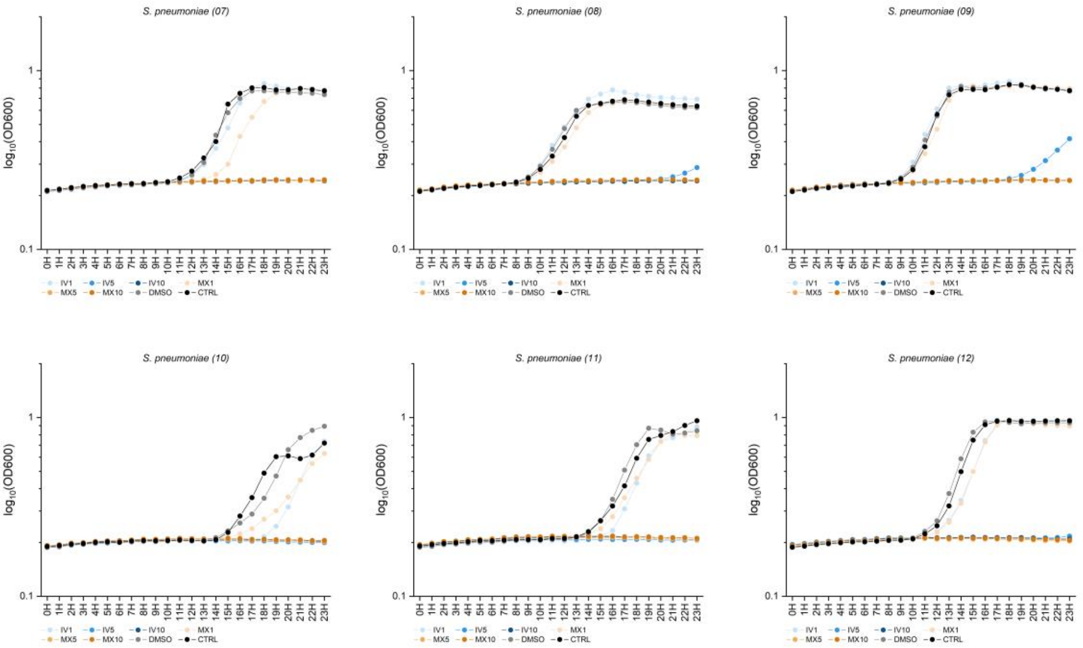

Supplementary Figure 7: Anaerobic growth curves of 6 bacterial isolates co-incubated with IV (blue) or MX (orange) at concentrations of either 1µM, 5µM or 10µM. Each datapoint represents an average of two independent experiments. The grey curves represent growth in presence of 0.2% DMSO, the black curve represents a positive growth control in presence of BHI + 5% yeast only. Left to right and top to bottom: S. pneumoniae (07), S. pneumoniae (08), S. pneumoniae (09), S. pneumoniae (10), S. pneumoniae (11) and S. pneumoniae (12).

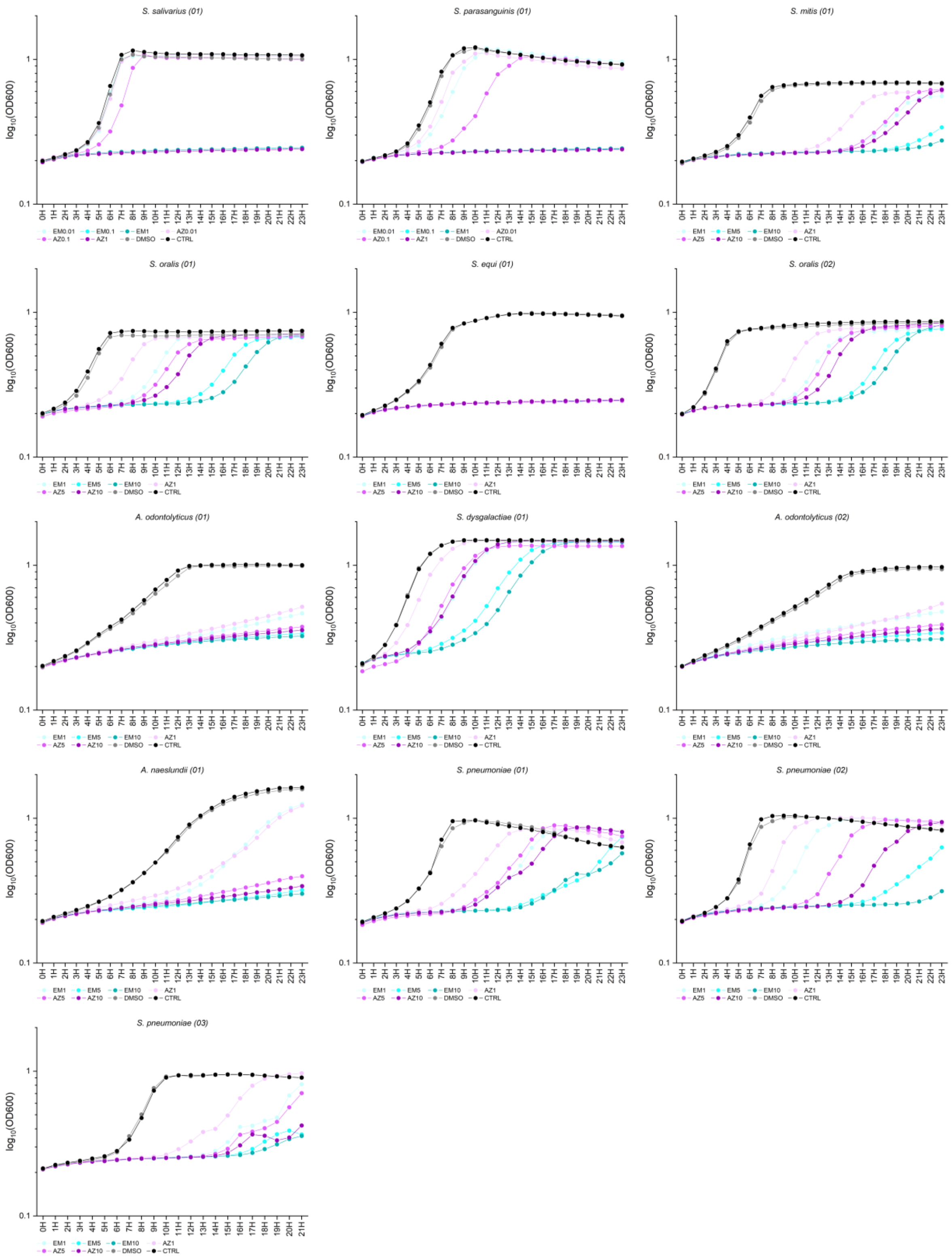

Supplementary Figure 8: Aerobic growth curves of 13 bacterial isolates co-incubated with EM (turquoise) or AZ (purple) at three different concentrations. Each datapoint represents an average of two independent experiments. The grey curves represent growth in presence of 0.2% DMSO, the black curve represents a positive growth control in presence of BHI + 5% yeast only. Left to right and top to bottom: S. salivarius (01), S. parasanguinis (01), S. mitis (01), S. oralis (01), S. equi (01), S. oralis (02), A. odontolyticus (01), S. dysgalactiae (01), A. odontolyticus (02), A. naeslundii (01), S. pneumoniae (01), S. pneumoniae (02) and S. pneumoniae (03).

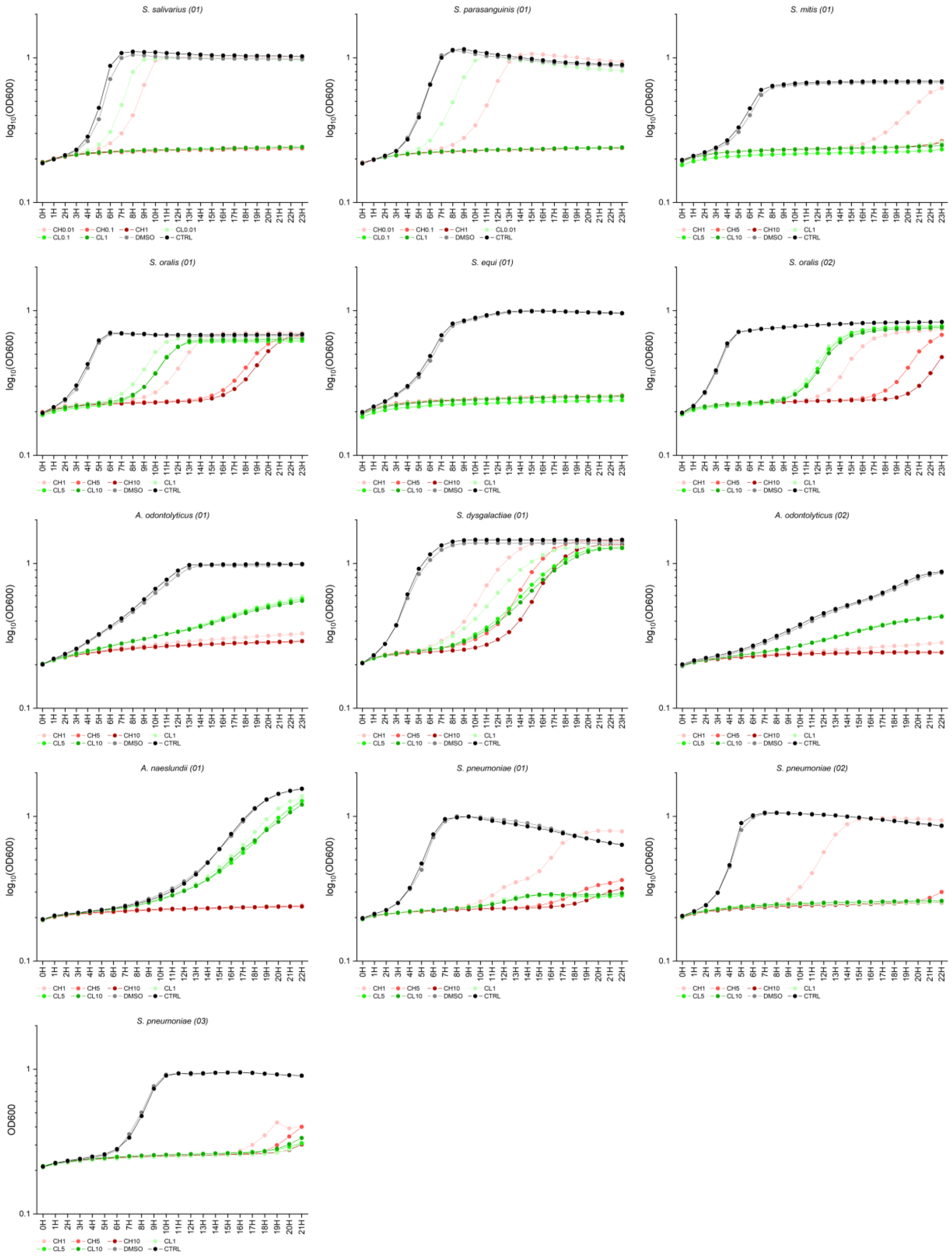

Supplementary Figure 9: Aerobic growth curves of 13 bacterial isolates co-incubated with CH (red) or CL (green) at three different concentrations. Each datapoint represents an average of two independent experiments. The grey curves represent growth in presence of 0.2% DMSO, the black curve represents a positive growth control in presence of BHI + 5% yeast only. Left to right and top to bottom: S. salivarius (01), S. parasanguinis (01), S. mitis (01), S. oralis (01), S. equi (01), S. oralis (02), A. odontolyticus (01), S. dysgalactiae (01), A. odontolyticus (02), A. naeslundii (01), S. pneumoniae (01), S. pneumoniae (02) and S. pneumoniae (03).

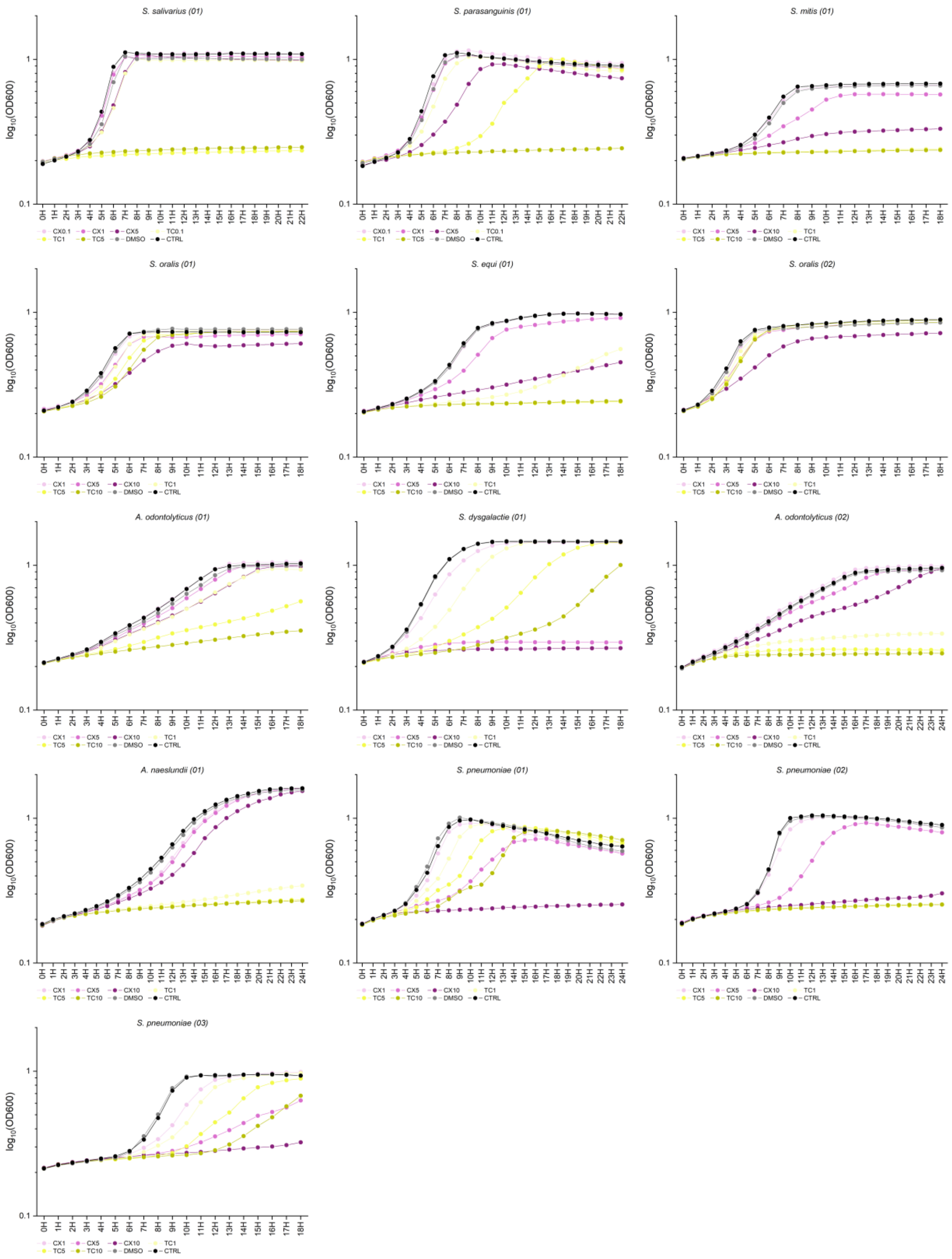

Supplementary Figure 10: Aerobic growth curves of 13 bacterial isolates co-incubated with CX (mauve) or TC (yellow) at three different concentrations. Each datapoint represents an average of two independent experiments. The grey curves represent growth in presence of 0.2% DMSO, the black curve represents a positive growth control in presence of BHI + 5% yeast only. Left to right and top to bottom: S. salivarius (01), S. parasanguinis (01), S. mitis (01), S. oralis (01), S. equi (01), S. oralis (02), A. odontolyticus (01), S. dysgalactiae (01), A. odontolyticus (02), A. naeslundii (01), S. pneumoniae (01), S. pneumoniae (02) and S. pneumoniae (03).

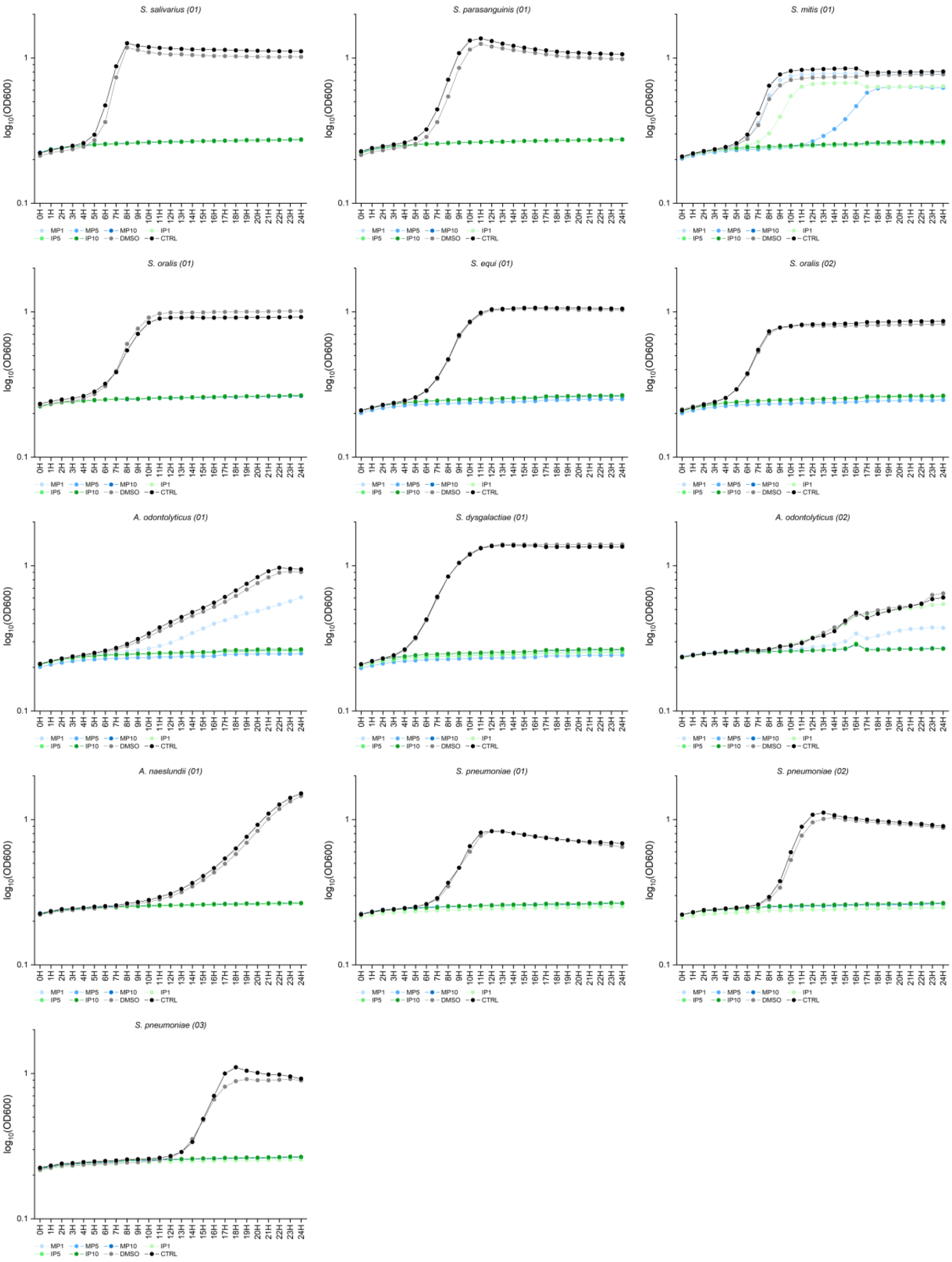

Supplementary Figure 11: Aerobic growth curves of 13 bacterial isolates co-incubated with MP (blue) or IP (green) at three different concentrations. Each datapoint represents an average of two independent experiments. The grey curves represent growth in presence of 0.2% DMSO, the black curve represents a positive growth control in presence of BHI + 5% yeast only. Left to right and top to bottom: S. salivarius (01), S. parasanguinis (01), S. mitis (01), S. oralis (01), S. equi (01), S. oralis (02), A. odontolyticus (01), S. dysgalactiae (01), A. odontolyticus (02), A. naeslundii (01), S. pneumoniae (01), S. pneumoniae (02) and S. pneumoniae (03).

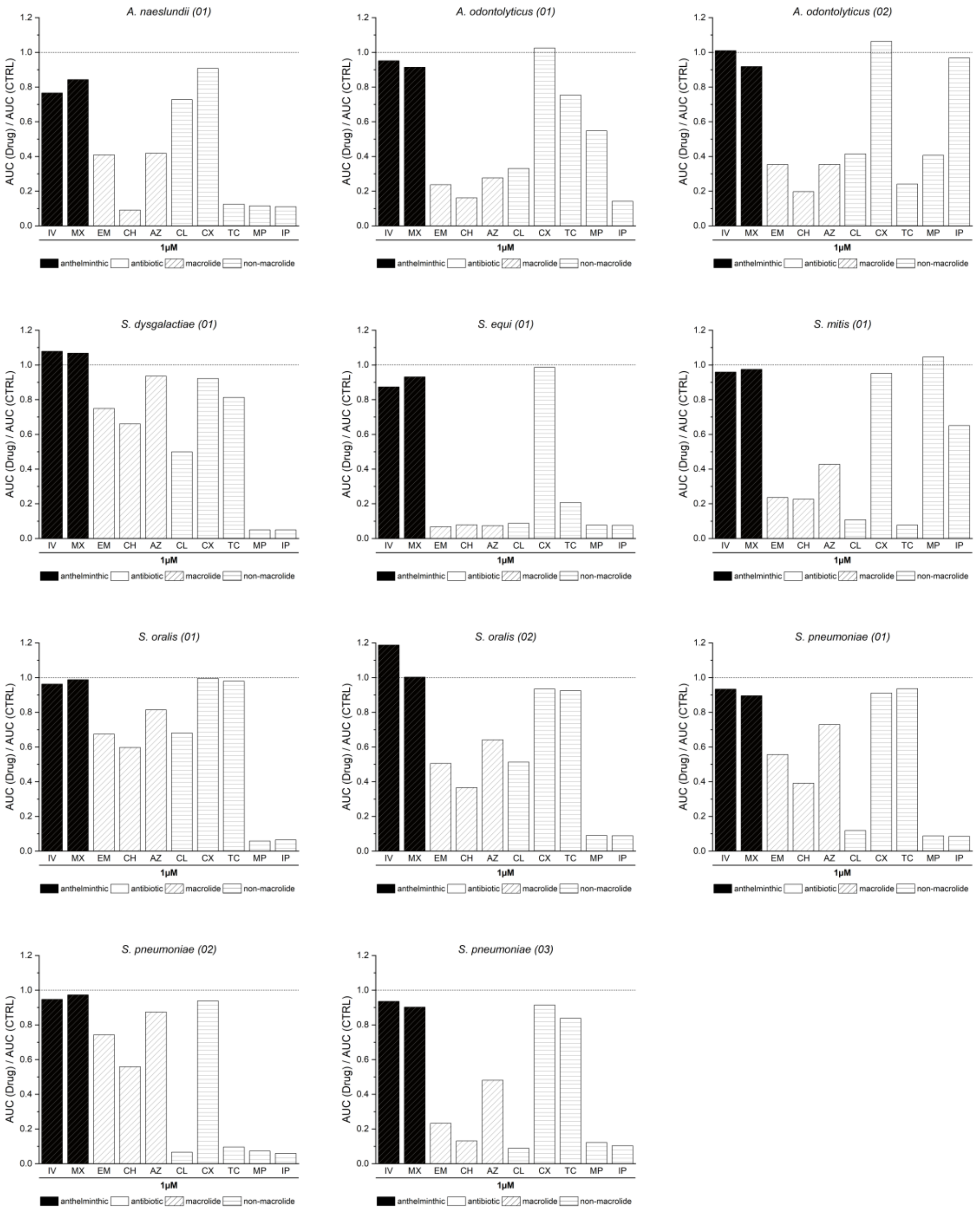

Supplementary Figure 12: AUC ratios of 11 bacterial isolates in presence of IV, MX, EM, CH, AZ, CL, CX, TC, MP or IP at 1µM. The dotted line marks theoretical uninhibited growth of the isolate (AUC ratio = 1). Bar colors correspond to the primary usecase of the compound. Black = anthelminthic, white = antibiotic.

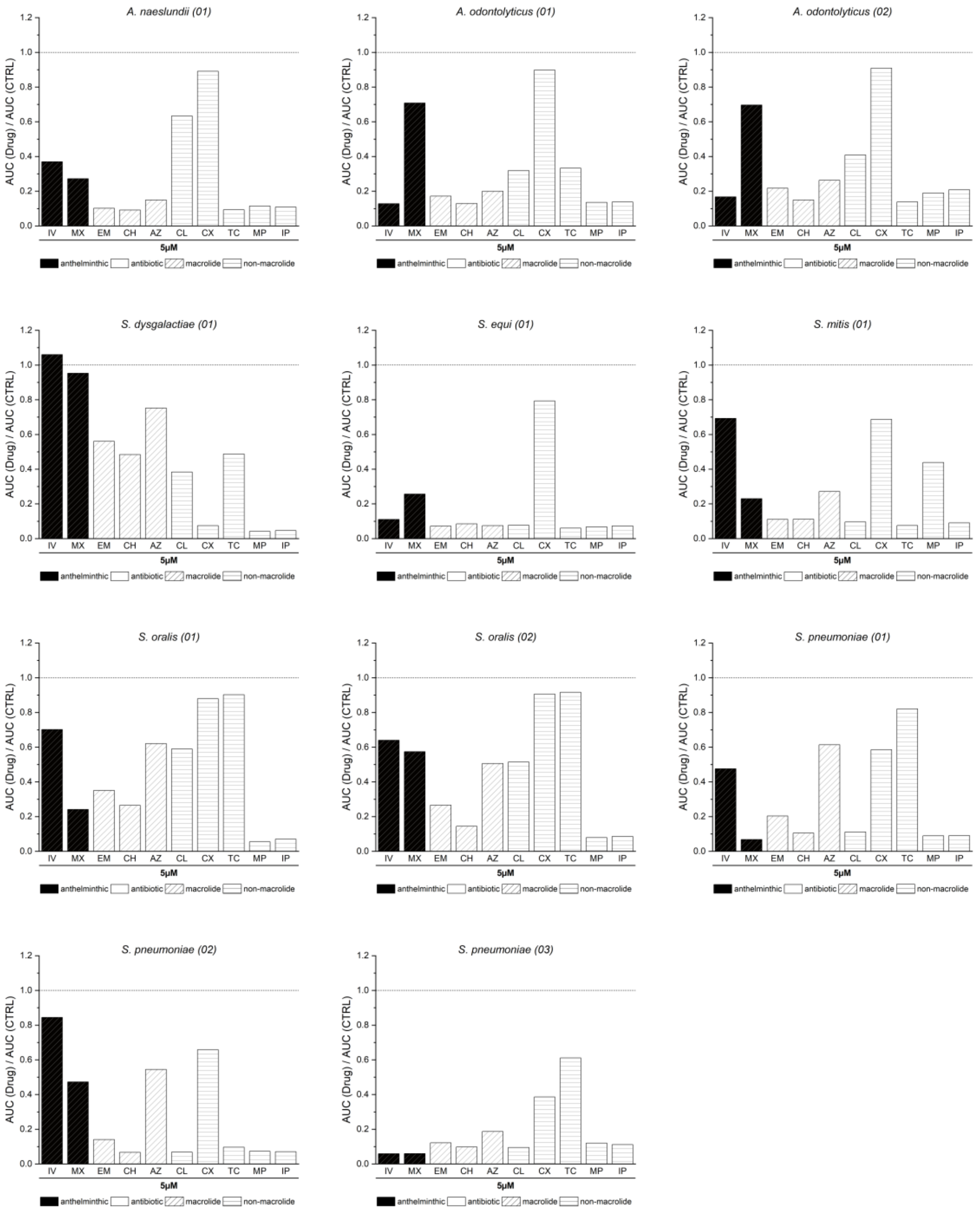

Supplementary Figure 13: AUC ratios of 11 bacterial isolates in presence of IV, MX, EM, CH, AZ, CL, CX, TC, MP or IP at 5µM. The dotted line marks theoretical uninhibited growth of the isolate (AUC ratio = 1). Bar colors correspond to the primary usecase of the compound. Black = anthelminthic, white = antibiotic.

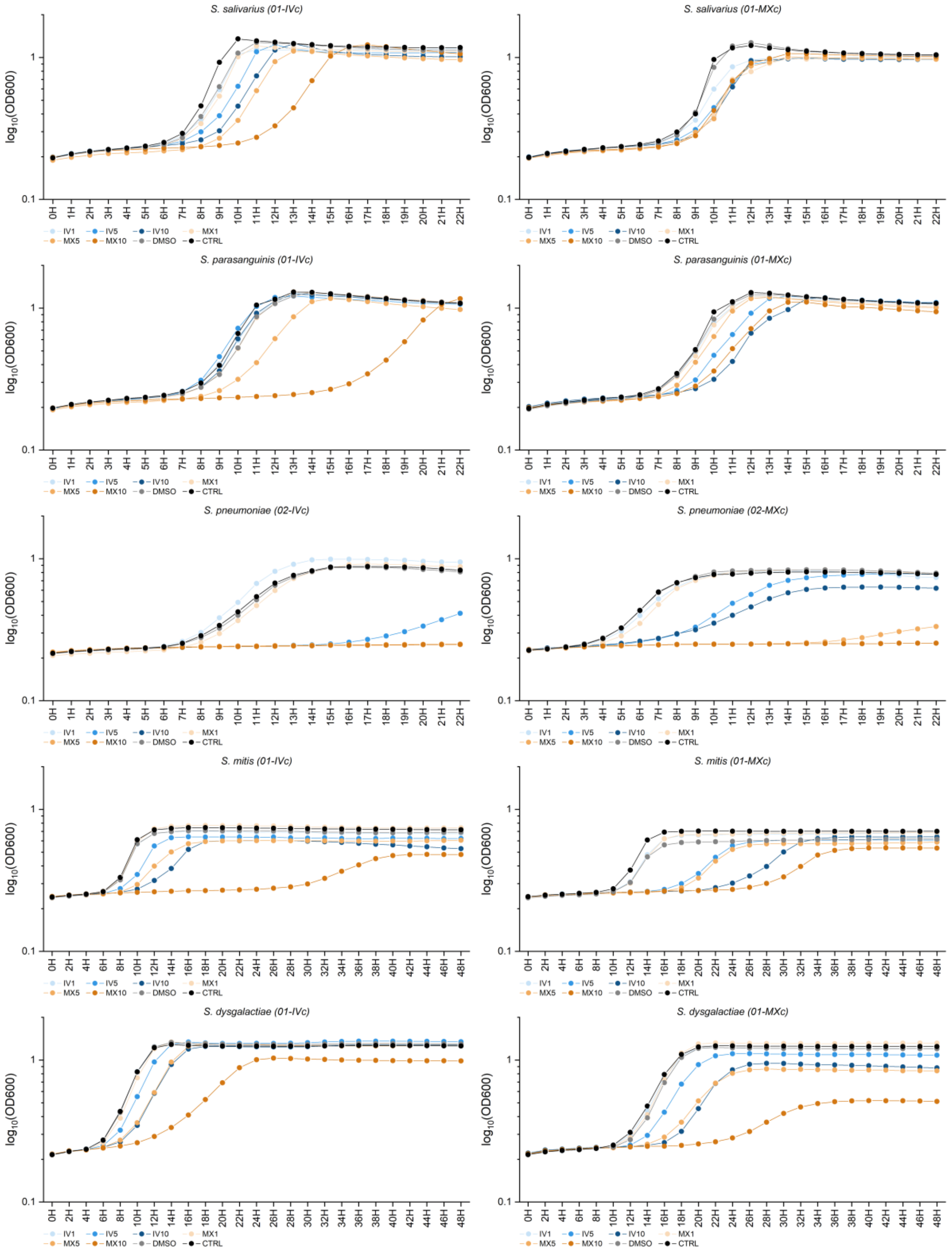

Supplementary Figure 14: Aerobic growth curves of 10 anthelminthic-prechallenged bacterial isolates co-incubated with IV (blue) or MX (orange) at either 1µM, 5µM or 10µM. Each datapoint represents an average of two independent experiments. The grey curves represent growth in presence of 0.2% DMSO, the black curve represents a positive growth control in presence of BHI + 5% yeast only. Left to right and top to bottom: S. salivarius (01-IVc), S. salivarius (01-MXc), S. parasanguinis (01-IVc), S. parasanguinis (01-MXc), S. pneumoniae (02-IVc), S. pneumoniae (02-MXc), S. mitis (01-IVc), S. mitis (01-MXc), S. dysgalactiae (01-IVc) and S. dysgalactiae (01-MXc).

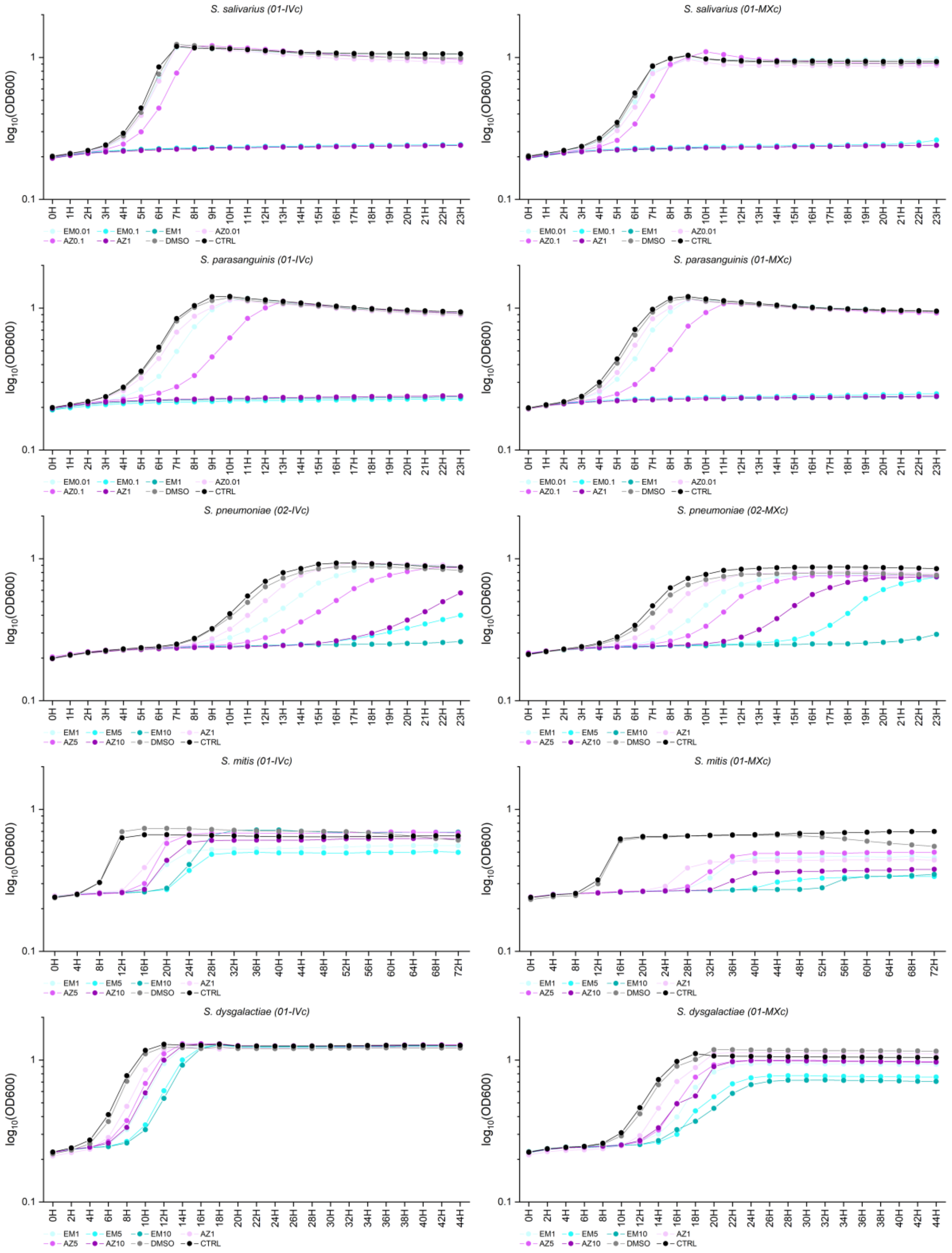

Supplementary Figure 15: Aerobic growth curves of 10 anthelminthic-prechallenged bacterial isolates co-incubated with EM (turquoise) or AZ (purple) at three different concentrations. Each datapoint represents an average of two independent experiments. The grey curves represent growth in presence of 0.2% DMSO, the black curve represents a positive growth control in presence of BHI + 5% yeast only. Left to right and top to bottom: S. salivarius (01-IVc), S. salivarius (01-MXc), S. parasanguinis (01-IVc), S. parasanuinis (01-MXc), S. pneumoniae (02-IVc), S. pneumoniae (02-MXc), S. mitis (01-IVc), S. mitis (01-MXc), S. dysgalactiae (01-IVc) and S. dysgalactiae (01-MXc).

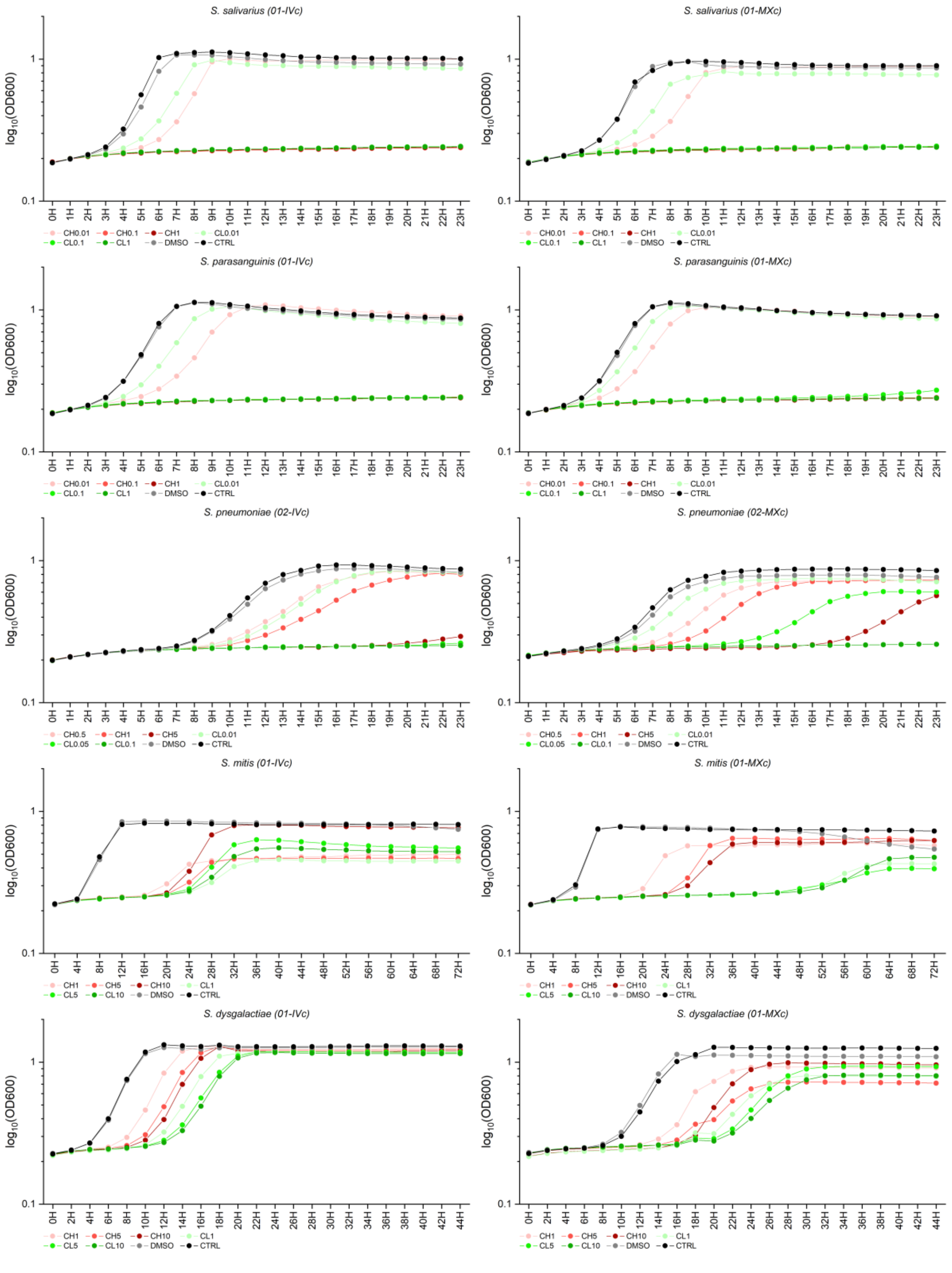

Supplementary Figure 16: Aerobic growth curves of 10 anthelminthic-prechallenged bacterial isolates co-incubated with CH (red) or CL (green) at three different concentrations. Each datapoint represents an average of two independent experiments. The grey curves represent growth in presence of 0.2% DMSO, the black curve represents a positive growth control in presence of BHI + 5% yeast only. Left to right and top to bottom: S. salivarius (01-IVc), S. salivarius (01-MXc), S. parasanguinis (01-IVc), S. parasanuinis (01-MXc), S. pneumoniae (02-IVc), S. pneumoniae (02-MXc), S. mitis (01-IVc), S. mitis (01-MXc), S. dysgalactiae (01-IVc) and S. dysgalactiae (01-MXc).

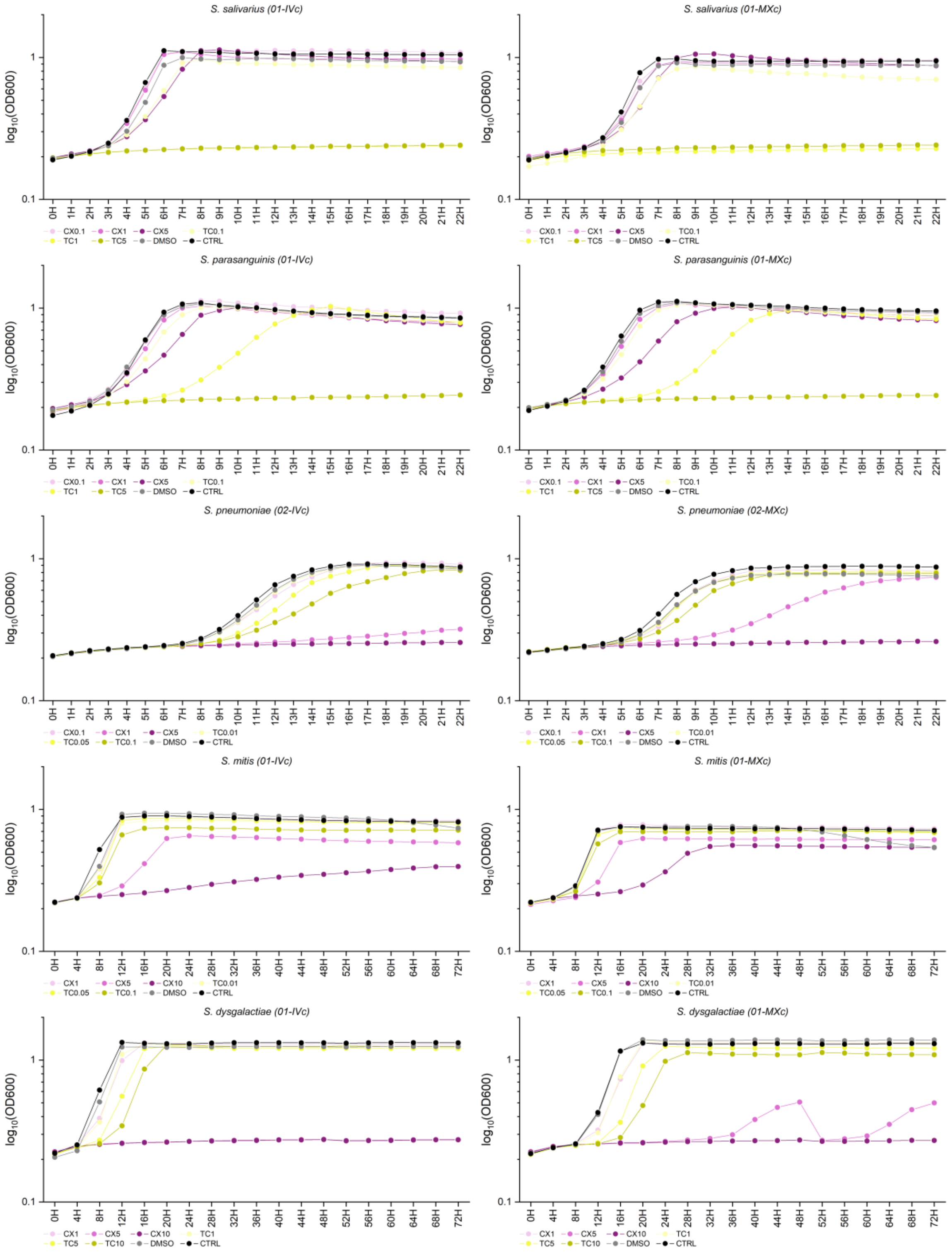

Supplementary Figure 17: Aerobic growth curves of 10 anthelminthic-prechallenged bacterial isolates co-incubated with CX (mauve) or TC (yellow) at three different concentrations. Each datapoint represents an average of two independent experiments. The grey curves represent growth in presence of 0.2% DMSO, the black curve represents a positive growth control in presence of BHI + 5% yeast only. Left to right and top to bottom: S. salivarius (01-IVc), S. salivarius (01-MXc), S. parasanguinis (01-IVc), S. parasanuinis (01-MXc), S. pneumoniae (02-IVc), S. pneumoniae (02-MXc), S. mitis (01-IVc), S. mitis (01-MXc), S. dysgalactiae (01-IVc) and S. dysgalactiae (01-MXc).

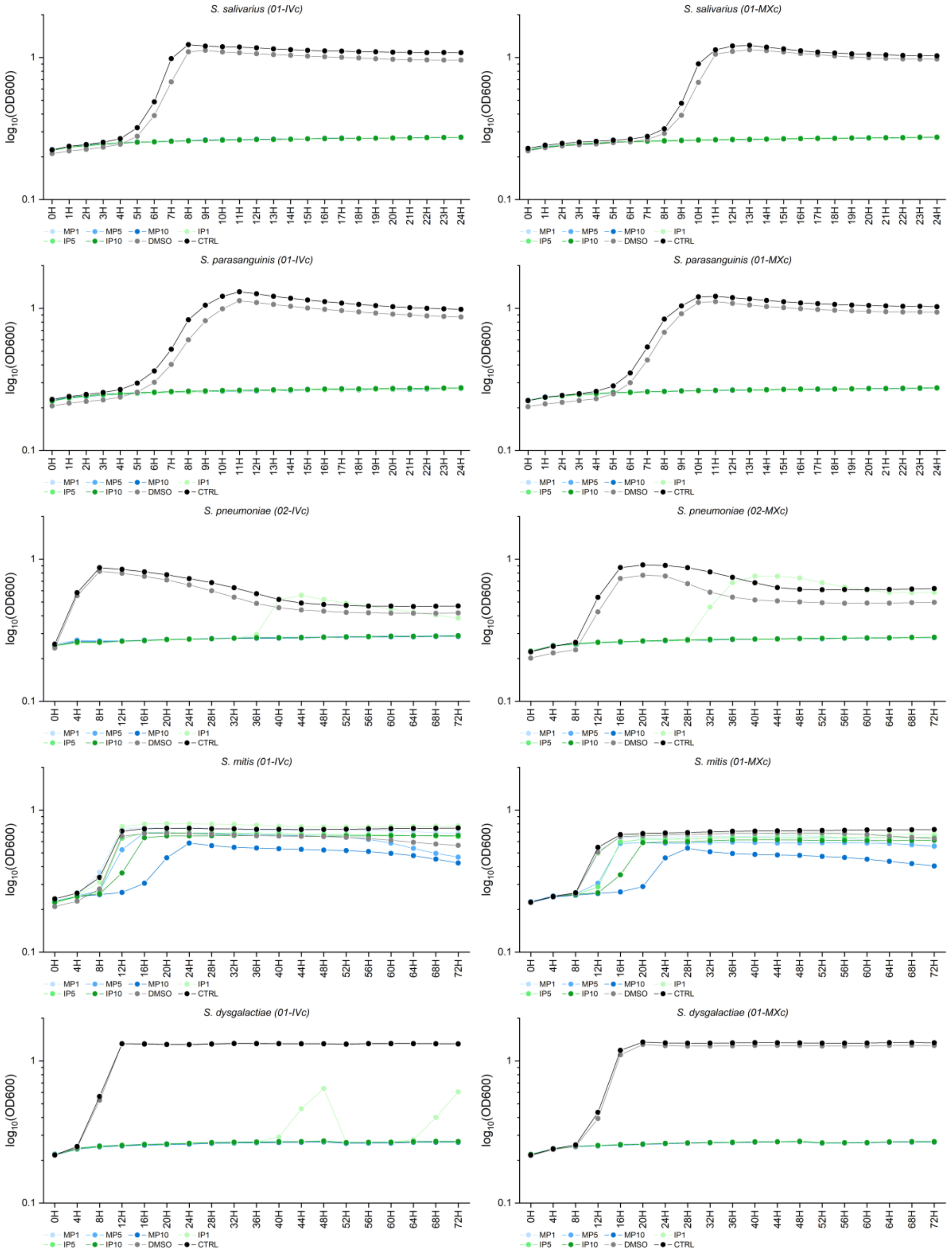

Supplementary Figure 18: Aerobic growth curves of 10 anthelminthic-prechallenged bacterial isolates co-incubated with MP (blue) or IP (green) at three different concentrations. Each datapoint represents an average of two independent experiments. The grey curves represent growth in presence of 0.2% DMSO, the black curve represents a positive growth control in presence of BHI + 5% yeast only. Left to right and top to bottom: S. salivarius (01-IVc), S. salivarius (01-MXc), S. parasanguinis (01-IVc), S. parasanuinis (01-MXc), S. pneumoniae (02-IVc), S. pneumoniae (02-MXc), S. mitis (01-IVc), S. mitis (01-MXc), S. dysgalactiae (01-IVc) and S. dysgalactiae (01-MXc).
