# Supplementary material for: Exposure of gut bacterial isolates to the anthelminthic drugs, ivermectin and moxidectin, leads to antibiotic-like phenotypes of growth inhibition and adaptation
Source: Commun Biol. 2024 Nov 25;7:1566. doi: 10.1038/s42003-024-07135-z (PMC11603213; doi:10.1038/s42003-024-07135-z)
Supplement: Supplementary file 3 — Reporting-summary [file 42003_2024_7135_MOESM3_ESM.pdf]

nature portfolio

Corresponding author(s): Pierre Schneeberger

Last updated by author(s): Oct 17, 2024

Reporting Summary

Nature Portfolio wishes to improve the reproducibility of the work that we publish. This form provides structure for consistency and transparency in reporting. For further information on Nature Portfolio policies, see our Editorial Policies and the Editorial Policy Checklist.

Statistics

For all statistical analyses, confirm that the following items are present in the figure legend, table legend, main text, or Methods section.

n/a	Confirmed	
☐	☒	The exact sample size (*n*) for each experimental group/condition, given as a discrete number and unit of measurement
☐	☒	A statement on whether measurements were taken from distinct samples or whether the same sample was measured repeatedly
☐	☒	The statistical test(s) used AND whether they are one- or two-sided<br>*Only common tests should be described solely by name; describe more complex techniques in the Methods section.*
☐	☒	A description of all covariates tested
☐	☒	A description of any assumptions or corrections, such as tests of normality and adjustment for multiple comparisons
☒	☐	A full description of the statistical parameters including central tendency (e.g. means) or other basic estimates (e.g. regression coefficient) AND variation (e.g. standard deviation) or associated estimates of uncertainty (e.g. confidence intervals)
☐	☒	For null hypothesis testing, the test statistic (e.g. *F*, *t*, *r*) with confidence intervals, effect sizes, degrees of freedom and *P* value noted<br>*Give P values as exact values whenever suitable.*
☒	☐	For Bayesian analysis, information on the choice of priors and Markov chain Monte Carlo settings
☒	☐	For hierarchical and complex designs, identification of the appropriate level for tests and full reporting of outcomes
☐	☒	Estimates of effect sizes (e.g. Cohen's *d*, Pearson's *r*), indicating how they were calculated

Our web collection on statistics for biologists contains articles on many of the points above.

Software and code

Policy information about availability of computer code

Data collection	We utilized a Hidex Sense plate reader (Hidex Oy, Turku, Finland) to conduct OD600 measurements. Data collection was handled by Hidex' built in Hidex Sense software (v. 1.2.1).
Data analysis	Data of bacterial growth curves, as well as area under the curve (AUC) ratios were curated in Microsoft Excel 2016 and plotted using OriginPro 2022b (OriginLab Corporation, Northhampton MA, United States). To calculate AUC ratios the R package growthcurver [42] (v0.3.1) was used in RStudio equipped with R 4.1.3. Pairwise Wilcoxon rank sum exact tests and a kruskal wallis tests where calculated in RStudio equipped with R 4.1.3. The Pearson correlation matrix and p-values for averaged duplicate AUC ratio values of 11 bacterial isolates in presence of IV/MX or macrolides/lincosamide antibiotics were calculated in Microsoft Excel 2016.

For manuscripts utilizing custom algorithms or software that are central to the research but not yet described in published literature, software must be made available to editors and reviewers. We strongly encourage code deposition in a community repository (e.g. GitHub). See the Nature Portfolio guidelines for submitting code & software for further information.

Data

Policy information about availability of data

All manuscripts must include a data availability statement. This statement should provide the following information, where applicable:

- Accession codes, unique identifiers, or web links for publicly available datasets
- A description of any restrictions on data availability
- For clinical datasets or third party data, please ensure that the statement adheres to our policy

The sequencing data (16S rRNA gene sequencing) generated in this study have been deposited in the NCBI Short Read Archive under the accession PRJNA1053597. The numerical source data underlying all figures have been deposited under this DOI: https://doi.org/10.6084/m9.figshare.27248703.v1

Human research participants

Policy information about studies involving human research participants and Sex and Gender in Research.

Reporting on sex and gender	see DOI: 10.1016/S1473-3099(21)00421-7
Population characteristics	see DOI: 10.1016/S1473-3099(21)00421-7
Recruitment	see DOI: 10.1016/S1473-3099(21)00421-7
Ethics oversight	see DOI: 10.1016/S1473-3099(21)00421-7

Note that full information on the approval of the study protocol must also be provided in the manuscript.

Field-specific reporting

Please select the one below that is the best fit for your research. If you are not sure, read the appropriate sections before making your selection.

☒ Life sciences ☐ Behavioural & social sciences ☐ Ecological, evolutionary & environmental sciences

For a reference copy of the document with all sections, see nature.com/documents/nr-reporting-summary-flat.pdf

Life sciences study design

All studies must disclose on these points even when the disclosure is negative.

Sample size	No sample sizes were calculated. Bacterial isolates were selected as follows:<br>20/59 bacterial isolates are macrolide or lincosamide resistant and were obtained via the Institute for Infectious Diseases (Berne, Switzerland). As lincosamides share the mode of action with macrolides, resistance mechanisms often overlap. We therefore included these isolates to test whether macrolide or lincosamide resistance is further coupled to sensitivity to IV/MX. The lincosamide resistant strains were comprised of 5 Streptococcus spp. and 3 Actinomyces spp. Streptococcus species are frequent, gram-positive commensals in the oral microbiota, but are especially abundant in the small intestine. However, several mechanisms of macrolide resistance have been identified among Streptococcus species, including mainly drug efflux, target alteration, and drug inactivation. Moreover, Streptococcus salivarius – amongst others – was associated with ALB-IV combination treatment failure in a recent study (Schneeberger et al. Nat Commun, 2022). Actinomyces spp. on the other hand are close relatives to avermectin producing bacteria (Streptomyces avermitilis). They therefore likely possessed unique co-incubation phenotypes with derivatives such as IV or MX. The macrolide resistant isolates were comprised solely of S. pneumoniae isolates (n = 12). S. pneumoniae is primarily found in the lungs but also commonly found throughout the upper and lower GI tract, and is one of the six leading pathogens causing lethal lower respiratory infections. We included 27/59 bacterial isolates from clinical stool samples from a recent study conducted in Lao PDR (NCT03527732). As Lao PDR represents a prime site of clinical trials involving both IV and MX and lies in midst of a hotspot of helminth prevalence, the corresponding bacterial isolates likely reflect an extensive clinical background. Bacterial isolation from stool samples was either conducted at Swiss TPH in Allschwil, Switzerland (18/59 bacterial isolates) or by Dr. Julian Garneau and Alison Gandelin at the University of Lausanne in Lausanne, Switzerland (9/59 bacterial isolates). Enriched clinical isolates were comprised of the genera Blautia, Clostridium, Dorea, Enterococcus, Escherichia and Streptococcus. It is critical to investigate this interaction with IV/MX in several commensal species as they might act as reservoir for AMR genes and thus contribute to the persistence of AMR in the human body. Therefore, we included 12/59 commercial isolates (Bacteroides, Blautia, Dorea, Lactobacillus, Staphylococcus, Streptococcus), to further broaden taxonomic range of our results. The commercial isolates were purchased from the German Collection of Microorganisms and Cell Cultures (https://www.dsmz.de/).
Data exclusions	No data were excluded from the analyses.
Replication	All bacterial growth cruves, as well as Etests were measured in duplicate.
Randomization	NA

Blinding	NA

Reporting for specific materials, systems and methods

We require information from authors about some types of materials, experimental systems and methods used in many studies. Here, indicate whether each material, system or method listed is relevant to your study. If you are not sure if a list item applies to your research, read the appropriate section before selecting a response.

Materials & experimental systems

n/a	Involved in the study	
☒	☐	Antibodies
☒	☐	Eukaryotic cell lines
☒	☐	Palaeontology and archaeology
☒	☐	Animals and other organisms
☐	☒	Clinical data
☒	☐	Dual use research of concern

Methods

n/a	Involved in the study	
☒	☐	ChIP-seq
☒	☐	Flow cytometry
☒	☐	MRI-based neuroimaging

Clinical data

Policy information about clinical studies

All manuscripts should comply with the ICMJE guidelines for publication of clinical research and a completed CONSORT checklist must be included with all submissions.

Clinical trial registration	NCT03527732
Study protocol	see DOI: 10.1016/S1473-3099(21)00421-7
Data collection	see DOI: 10.1016/S1473-3099(21)00421-7
Outcomes	see DOI: 10.1016/S1473-3099(21)00421-7
